# Prof. Davis’ Clinique in the Medical Department of Lind University

**Published:** 1860-07

**Authors:** 


### PROF. DAVIS' CLINIQUE IN THE MEDICAL DEPARTMENT OF LIND UNIVERSITY.

Saturday, June 23d, 1860.

Case 1st. *General Irritation of the Mucous Membranes.*—This patient is a child aged 10 months. The mother says it has been feverish and restless all the past week. It has a frequent, harrassing cough, with some rattling of mucous in the air passages; together with frequent discharges from the bowels. The fœces are thin, variable in color, and mixed with mucus. The stomach is also sensitive, often rejecting its milk after nursing, and crying as if troubled with colic pains. On looking into the mouth you find the mucous lining redder than natural, more hot, as well as more heat of the cutaneous surface generally.

From these symptoms we infer that a low grade of inflammation exists in the mucous membrane of the air passages and the lower half of the bowels. Such cases have been frequently met with during the last few days, and in most instances the following mixture has afforded the most prompt relief:

℞ Ol. Terebrinth, ʒ i.<br>

Tinct. Opii. ʒ i.<br>

Pulv. G. Arabac, } a.a. ʒ iii.<br>

White Sugar, }

Rub together, and add:

Comp. Honey of Squills, &c. ʒ iii.<br>

Mint Water, ℥ iss.

Mix, and give the child from twenty to thirty drops every three or four hours.

We shall give the child this mixture, and request the mother to bring it again in two or three days if it is not fully relieved.

Case 2. *Hooping-Cough.*—This little child is only four months old. We are told that it has had a severe cough for two weeks past. The cough comes in distinct paroxysms, apparently very severe, and often ending with so much strangling or spasmodic difficulty of inspiration as to cause the face to become very turgid with blood, and the eyelids swollen.

Sometimes the efforts at inspiration are accompanied by a loud stridulous sound called the *hoop.* This disease is distinguished from bronchitis or pneumonia by the distinctness and severity of the cough, the strangling that accompanies it, and the apparent entire freedom from either febrile symptoms or dyspnœa during the intervals between the paroxyms.

The child will cough with such violence as to alarm the parents at one moment, and in ten minutes afterwards be either laughing and playful, or asleep, breathing as easy as if in perfect health.

The *hooping-cough* is a spasmodic affection, doubtless depending on some peculiar morbid condition of the par vagum, or that portion of it which is distributed upon the respiratory organs. It seldom proves fatal unless by complication with bronchitis or pneumonia.

When the disease occurs in the middle of summer it is apt to become complicated with diarrhœa, especially if the ordinary expectorants are used in its treatment. As the cough is purely spasmodic, dependent on nervous irritation, our chief reliance in the treatment of it must be upon anti-spasmodics and anodynes. We shall direct for this little child the following mixture:

℞ Tincture Lobelia, ℥ ss.
Tinct. Cannabis Ind. ℥ ss.

Mix, and give the child 8 drops, four times a day. This dose may be increased or diminished according to its effects.

Case 3. *Chronic Bronchitis with Asthma.*—Mrs. C., aged 35 years, has had a severe harsh cough for several months. The cough is deep and rough, accompanied by a sense of soreness behind the lower half of the sternum, and a scanty opaque expectoration. She says she often has paroxysms of dyspnœa so severe that she cannot lie down at night, and feels constantly a tightness or sense of constriction across the chest. There is no fever, and only a slight degree of emaciation. By auscultation, a coarse dry ronchus was heard over both sides of the chest, with prolonged wheezing expiration, as though the bronchial tubes were contracted to such a degree that the air escaped with difficulty. There was no increased vibration of voice, and no dulness on percussion.

The members of the class were allowed to examine the patient with the stethescope, after which the diagnosis between tuberculosis and bronchitis was fully discussed. The patient was evidently affected with that grade of chronic inflammation of the mucous lining of the bronchial tubes which causes the membrane to become congested, thickened, and yet dryer than natural. Hence, the narrowing of the tubes, causing the sense of tightness in the chest, while the sensitive filiments of nerves distributed upon the membrane partake of the inflammation sufficiently to cause frequent harrassing cough, and at times paroxysms of dyspnœa, constituting one of the varieties of Asthma.

The first object in the treatment of such a case is to diminish the morbid sensitiveness of the mucous surface of the air passages, and promote a more abundant secretion. Perhaps no remedial agents will do this more efficiently than the combination of Opium and Tartrate of Antimony and Potash. We shall, therefore, give her the following:

℞ Pulv. Opii, 8 grs.

Tart. Ant. et Pot., 1 gr.

White Sugar, 30 grs.

Mix, and divide into eight powders, and give one each morning, noon, tea-time, and bed-time. When they have all been taken, open the bowels moderately by a dose of Castor Oil. We shall expect by that time, the cough to have become much less severe, and the expectoration more free; and if so, she may then take the following mixture:

℞ Fluid Ext. Lactuca, ℥ iss.
Tinct. Lobelia, ℥ ss.
Tinct. Cimicifuga Rac., ℥ i.

Mix, and take a teaspoonful four times a day.

This formula may be rendered more efficacious for many chronic cases, accompanied by considerable thickening of the bronchial mucous membrane, by adding to it ʒ iss of Iodide of Potassa.

Case 4. *Incipient Tuberculosis.*—This was a woman, aged 40 years, presenting all the usual symptoms and signs of pulmonary tuberculosis in the early period of its advancement.

The case was profitably examined by the class, but we have so recently published a full clinical lecture on tuberculous cases, that we will not occupy space with the present one.

